# Evaluating the efficacy of surgical and conservative approaches in mild autonomous cortisol secretion: a meta-analysis

**DOI:** 10.3389/fendo.2024.1399311

**Published:** 2024-07-17

**Authors:** Xingxiang Ren, Min Nan, Xiaomei Zhang

**Affiliations:** Department of Endocrinology, Peking University International Hospital, Beijing, China

**Keywords:** mild autonomous cortisol secretion, subclinical Cushing’s syndrome, subclinical hypercortisolism, mild cortisol autonomous secretion, adrenal incidentaloma, surgical treatment and adrenalectomy mild autonomous cortisol secretion, metabolic disorder, adrenalectomy

## Abstract

**Introduction:**

The management of Mild Autonomous Cortisol Secretion (MACS) remains a topic of debate among clinicians, with differing opinions on the effectiveness of surgical intervention compared to conservative treatment methods. This meta-analysis provides a comprehensive assessment of available literature to determine the most effective approach for treating this condition.

**Methods:**

On December 1, 2023, an exhaustive literature search of English databases Embase, PubMed, the Cochrane Library, Scopus, Web of Science, as well as the Chinese databases China HowNet, Wanfang Database, SinoMed Database, and Weipu Database using the keywords “Mild Autonomous Cortisol Secretion”, “Subclinical Cushing’s Syndrome”, “Subclinical Hypercortisolism”, “Mild Cortisol Autonomous Secretion”, “Adrenal Incidentaloma”, “Surgical Treatment” and “Adrenalectomy”. The data were statistically analyzed using STATA version 15.0.

**Results:**

In this comprehensive analysis involving 629 patients with MACS, the therapeutic efficacy of adrenalectomy was evident. The meta-analysis results indicate that compared to conservative treatment, surgical intervention more effectively improves obesity indicators in patients: waist circumference (SMD=-0.62, 95% CI: -1.06 to -0.18), BMI (SMD=-0.41, 95% CI: -0.62 to -0.20), enhances glycemic control: fasting blood glucose (SMD=-0.47, 95% CI: -0.68 to -0.26), glycated hemoglobin (SMD=-0.66, 95% CI: -0.95 to -0.38), improves lipid metabolism: triglycerides (SMD=-0.45, 95% CI: -0.73 to -0.16), lowers blood pressure: systolic blood pressure (SMD=-1.04, 95% CI: -1.25 to -0.83), diastolic blood pressure (SMD=-0.89, 95% CI: -1.12 to -0.65), and ameliorates hormonal metabolic disorder: 24h urinary free cortisol (SMD=-1.10, 95% CI: -1.33 to -0.87), ACTH (SMD=2.30, 95% CI: 1.63 to 2.97). All these differences are statistically significant.

**Conclusion:**

This meta-analysis shows that, compared to conservative treatment, surgical treatment is more effective in improving obesity indicators, glycemic control, lipid metabolism, reducing blood pressure, and ameliorating hormonal metabolic disorders in patients with MACS. These statistically significant results highlight the importance of considering surgical intervention in the management of patients with MACS.

**Systematic review registration:**

https://www.crd.york.ac.uk/prospero, identifier CRD42023492527.

## Introduction

Mild Autonomous Cortisol Secretion (MACS) is characterized by an autonomous elevation of cortisol levels within the patient’s body, yet it lacks the overt clinical manifestations typically associated with classical Cushing’s Syndrome. The diagnostic challenge lies in the mild or indistinct symptoms, necessitating a comprehensive biochemical evaluation for accurate diagnosis (1, 2). The diagnostic cut-offs of MACS based on cortisol values after dexamethasone suppression test (DST) are controversial, The ESE-ENSAT (European Society of Endocrinology-European Network for the Study of Adrenal Tumors) recommended cortisol>1.8μg/dL (50 nmol/L) after 1 mg-DST rather than 5 μg/dL (138nmol/L) to define MACS (3–6). With the advancement of imaging techniques, the incidence of adrenal incidentalomas has been progressively increasing. Approximately 5-20% of patients with adrenal incidentalomas exhibit MACS, characterized by subtle alterations in the hypothalamic-pituitary-adrenal axis secretion (1, 2, 7). MACS is associated with osteoporosis, mood alterations, hypertension, abnormalities in glucose and lipid metabolism, increased incidence of cardiovascular diseases, and a higher mortality rate (8–12).

Presently, the comprehension and insights into MACS are in their nascent stages. Research indicates potential therapeutic benefits of adrenalectomy in ameliorating comorbidities associated with MACS. However, the paucity of extensive, randomized controlled trials and the lack of comprehensive long-term postoperative follow-up data hinder the establishment of the unequivocal efficacy of surgical intervention. Consequently, the net clinical advantage of surgical management in MACS continues to be a subject of ongoing medical debate (12–16).

## Methods

**Figure 1:**
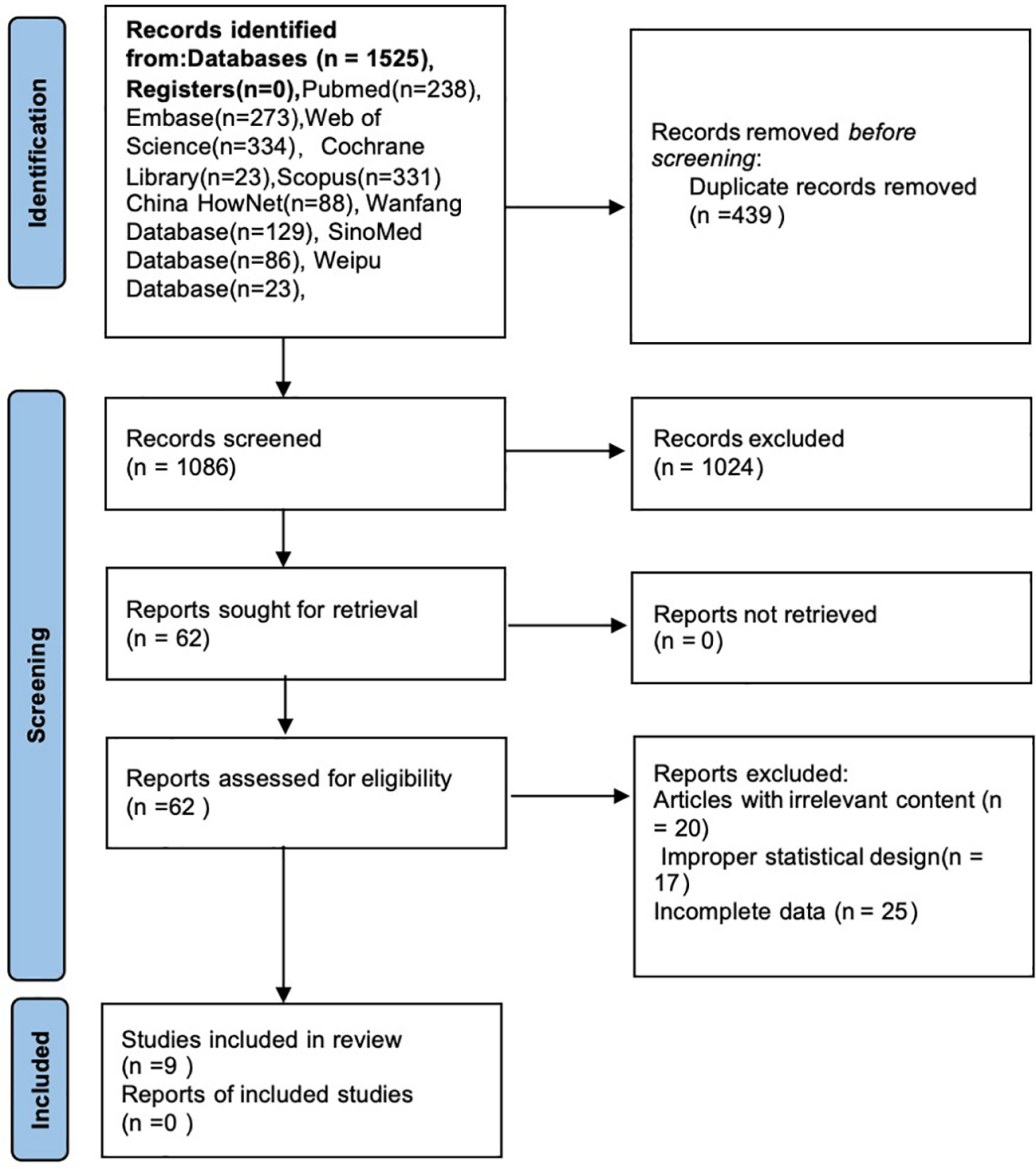
Flow diagram of the review process.

On December 1, 2023, an exhaustive literature search of English databases Embase, PubMed, the Cochrane Library, Scopus, Web of Science, as well as the Chinese databases China HowNet, Wanfang Database, SinoMed Database, and Weipu Database using the keywords “Subclinical Cushing’s Syndrome”, “Mild Autonomous Cortisol Secretion”, “Adrenal Incidentaloma”, “Surgical Treatment” and “Adrenalectomy”. A flow-chart of the literature review process is shown in **Figure 1**. The protocol for this research was registered on the PROSPERO platform (CRD42023492527).

Based on the latest guidelines published by the European Society of Endocrinology (ESE) in 2023, the diagnosis of Mild Autonomous Cortisol Secretion (MACS) primarily relies on the 1mg Dexamethasone Suppression Test (1mg-DST). Specifically, if serum cortisol levels fail to suppress below 1.8 µg/dL (50 nmol/L) after the 1mg dexamethasone suppression test, MACS can be considered (E-ENM) (ESE Home). Additionally, these patients typically do not exhibit the classic clinical features of Cushing’s syndrome but may have an increased risk of cardiovascular and metabolic conditions, such as hypertension and type 2 diabetes (Endocrinology.org). The literature included in this study, summarizing the diagnostic criteria for MACS, is compiled in **Table 1**.

**Table 1:** Characteristics of the studies included in the meta-analysis.

Authors Year Country (ref)	Definition of hypercortisolism	surgical group			non-surgical group		
		Number	Age	follow-up period	Number	Age	follow-up period
Chiodini Iacopo et al. 2009 Italy (11)	MACS defined as Serum cortisol levels after 1 mg dexamethasone suppression test (DST) greater than 3.0*µ*g/dl (83 nmol/liter)	25	54.8 ± 11.6	29.4 ± 13.8	16	64.4 ± 10.1	36.4 ± 11.6
Zhang W et al. 2007 China (13)	MACS defined as at least one of the three indexes of urine free cortisol, blood cortisol or circadian rhythm disturbance was abnormal at 24h, and the low-dose dexamethasone inhibition test could not inhibit it.	11	49.3 ± 9.0	25.0 ± 11.3	37	52.2 ± 8.9	25.0 ± 11.3
Liu MS et al. 2020 China (14)	MACS defined as Serum cortisol levels >138 nmol/L at 8:00 AM following suppression with either overnight 1 mg-DST or standard 2 mg-DST.	31	51 ± 11	11.6 ± 7.2	11	59 ± 18	12.0(6.0, 24.0)
Sui H et al. 2019 China (15)	MACS defined as laboratory tests indicating more than two signs of hypothalamic-pituitary-adrenal axis dysfunction, including elevated serum cortisol, increased 24-hour urinary free cortisol, or disrupted diurnal rhythm of cortisol that meets one of the criteria. Additionally, the low-dose dexamethasone suppression test does not show suppression.	18	46.4 ± 6.5	12	20	47.8 ± 7.6	12
Wang D et al. 2017 China (16)	MACS defined as at 8:00 AM, measurements of serum adrenocorticotropic hormone (ACTH), 24-hour urinary-free cortisol (UFC), and the 1 mg overnight dexamethasone suppression test (1 mg-DST) are conducted. At least one abnormal result among these three tests is required for diagnosis, with the result of the 1 mg-DST being the primary basis.	48	52	32	39	53	30
Yilmaz N et al. 2021 Turkey (17)	Patients with a high cortisol level following 1 mg dexamethasone suppression test (DST) and suppressed ACTH level (<10 pg/mL) plus one more positive test result compatible with hypercortisolemia (high cortisol level after two days of 2 mg DST, high 24-hour urinary free cortisol (UFC) level, low dehydroepiandrosteronesulfate (DHEA-S), high late-night salivary cortisol, or midnight serum cortisol level), but no symptoms or signs compatible with Cushing’s syndrome were considered to have SH.	57	57(34-75)	21(2-126)	8	65(46-75)	48(10-88)
Wang D et al. 2018 China (18)	MACS defined as cortisol levels greater than 1.8*µ*g/dL after 1-mg DST.	48	51.8 ± 10.2	32.5 ± 10.6	39	53.2 ± 12.1	30.1 ± 13.1
Iacobone Maurizio et al. 2012 Italy (19)	MACS defined as morning serum cortisol levels greater than 5 *µ*g/dL after the administration of 1 mg of dexamethasone in the evening the day before; morning ACTH levels less than 10 pg/mL, and daily urinary-free cortisol (UFC) greater than 76 *µ*g/day.	20	57(36-78)	54 ± 34	15	58(39-75)	56 ± 37
Salcuni Antonio Stefano et al. 2016 Italy (20)	Diagnosed SH by the absence of signs and/or symptoms of cortisol excess (i.e. striae rubrae,moon facies, buffalo hump, and skin atrophy) and by the presence (in at least two out of three different estimations)of cortisol levels after 1 mg overnight dexamethasonesuppression (1 mg-DST)>5.0 μg/dl (138 nmol/l) or in the presence of greater than or equal to two out of the following alterations: 1 mg-DsT >3.0 μg/d (83nmol/1),adrenocorticotropic hormone (ACTH) levels <10 pg/ml(2.2 pmol/l),24h urinary free cortisol (UFC) levels>70 μg/24 h (193 nmol/24 h).	32	65.4 ± 7.05	39.9 ± 20.9	23	61.3 ± 8.7	27.7 ± 11.1

MACS, Mild autonomous cortisol secretion; SH, Subclinical hypercortisolism; DST, Dexamethasone suppression test.

### Study selection and data extraction

The inclusion criteria for this meta-analysis are as follows: 1.Study type: includes randomized controlled trials, cohort studies, and case-control studies. 2.Subjects: patients clearly diagnosed with MACS, at least including (i) no obvious clinical manifestations and signs of hypercortisolism, (ii) patients not suppressed by the 1mg dexamethasone suppression test. 3.Interventions: undergoing adrenalectomy or conservative treatment. 4.The articles should include laboratory test data before and after surgical treatment or before and after conservative treatment(expressed as a continuous numerical variable). Exclusion criteria: 1.Studies not in Chinese or English. 2.Duplicate publications. 3.Reviews, conference proceedings, case reports, and other types of studies. 4.Studies without a clear definition of MACS or without a clear diagnosis.

Two independent reviewers (Ren and Nan) read the literature, selected the studies that met the inclusion and exclusion criteria, and extracted the study data, including the type of study, date of publication, first author, sample size of each group, intervention measures, follow-up time, and main outcome indicators. In case of disagreement, a third investigator resolved it.

### Quality assessment

The quality of each included study was determined by referring to the Newcastle-Ottawa Scale (NOS).

### Meta-analysis

This meta-analysis was conducted using STATA software, version 15.0. The I² test and Q test were employed to assess the heterogeneity among the included studies. When the I²<50% and the P>0.1, the heterogeneity among the studies is considered to be low, allowing for the use of a fixed-effects model to combine the effect sizes. Otherwise, sensitivity analysis and subgroup analysis can be conducted to explore the sources of heterogeneity, or a random-effects model may be used to combine the effect sizes. Publication bias was assessed using a funnel plot and Egger’s test, with a p-value <0.05 considered statistically significant.

## Results

### Literature search, basic information, and quality assessments

After the search, a total of 1525 related articles were identified. After removing duplicates, 1086 articles remained. Excluding reviews, case reports, and conference papers left 862 articles. After screening abstracts for relevance, 62 articles remained. Upon full-text reading, articles with irrelevant content, improper statistical design, or those for which the full text could not be obtained were excluded, resulting in 9 (15, 17–24) articles being included in the final analysis. In these 9 articles, conservative treatment refers to oral drug treatment, including oral hypoglycemic drugs, antihypertensive drugs, lipid-lowering drugs and other drugs for hormonal abnormalities comorbidities, not at hypercortisolism itself (e.g.low-dose steroidogenesis inhibitor). The detailed screening process is shown in **Figure 1**. **Table 2** provides information on the included studies, all of which were scored using the NOS (Newcastle-Ottawa Scale) with scores of 6 or above, as detailed in **Table 2**. The characteristics of the studies included in the meta-analysis, including the diagnostic criteria for MACS, are summarized in **Table 1**.

**Table 2:** Basic information and quality evaluation of previous research.

Author	Year	Type	NOS			
			Selection	Comparability	Outcome	Scores
Zhang	2007	Case-control study	☆☆	☆☆	☆☆☆	7☆
Liu	2020	Case-control study	☆☆	☆☆	☆☆☆	7☆
Sui	2019	Cohort study	☆☆	☆☆	☆☆	6☆
Wang	2017	Case-control study	☆☆☆	☆☆	☆☆☆	8☆
Yilmaz	2021	Case-control study	☆☆☆	☆☆	☆☆☆	8☆
Wang	2018	Case-control study	☆☆☆	☆☆	☆☆☆	8☆
Salcuni	2016	Case-control study	☆☆☆	☆☆	☆☆☆	8☆
Iacobone	2012	Cohort study	☆☆☆	☆☆	☆☆	7☆
Chiodini	2009	Case-control study	☆☆☆	☆☆	☆☆☆	8☆

☆ indicates a score of one point in the evaluation.

### Changes in obesity indicators

**Figure 2:**
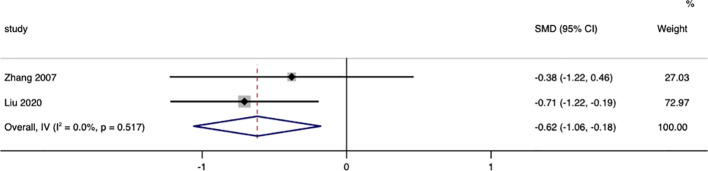
Waist Circumference-related forest.

Two studies were included that reported changes in waist circumference between surgical patients and those receiving conservative treatment. Heterogeneity testing resulted in I^2^ = 0.0%, p = 0.517, indicating very little heterogeneity in the selection of studies for this research. Therefore, a fixed-effects model was used to combine effect sizes. The results showed that, compared to the conservative treatment group, the change in waist circumference was more significant in the surgical group (SMD =-0.62, 95% CI: -1.06 to -0.18; p < 0.05) (**Figure 2**).

**Figure 3:**
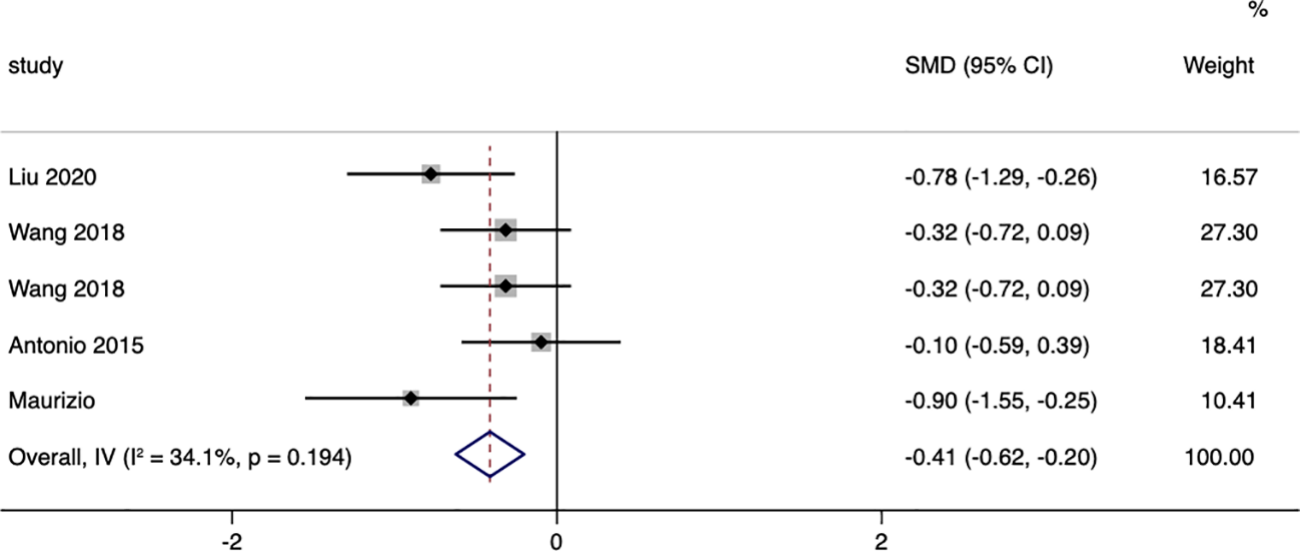
BMI-related forest.

Five studies were included that assessed changes in BMI between surgical patients and those receiving conservative treatment. After testing for heterogeneity, I^2^ = 34.1%, p=0.194, indicating very little heterogeneity in the selection of studies for this research. Therefore, a fixed-effects model was employed to combine effect sizes. The results showed a more significant reduction in BMI in the surgical group (SMD = -0.41, 95% CI: -0.62 to -0.20; p < 0.05). (**Figure 3**). The funnel plot (**Supplementary Figure S1**) and Egger’s test (p=0.624) showed no publication bias.

### Glucose metabolism

**Figure 4:**
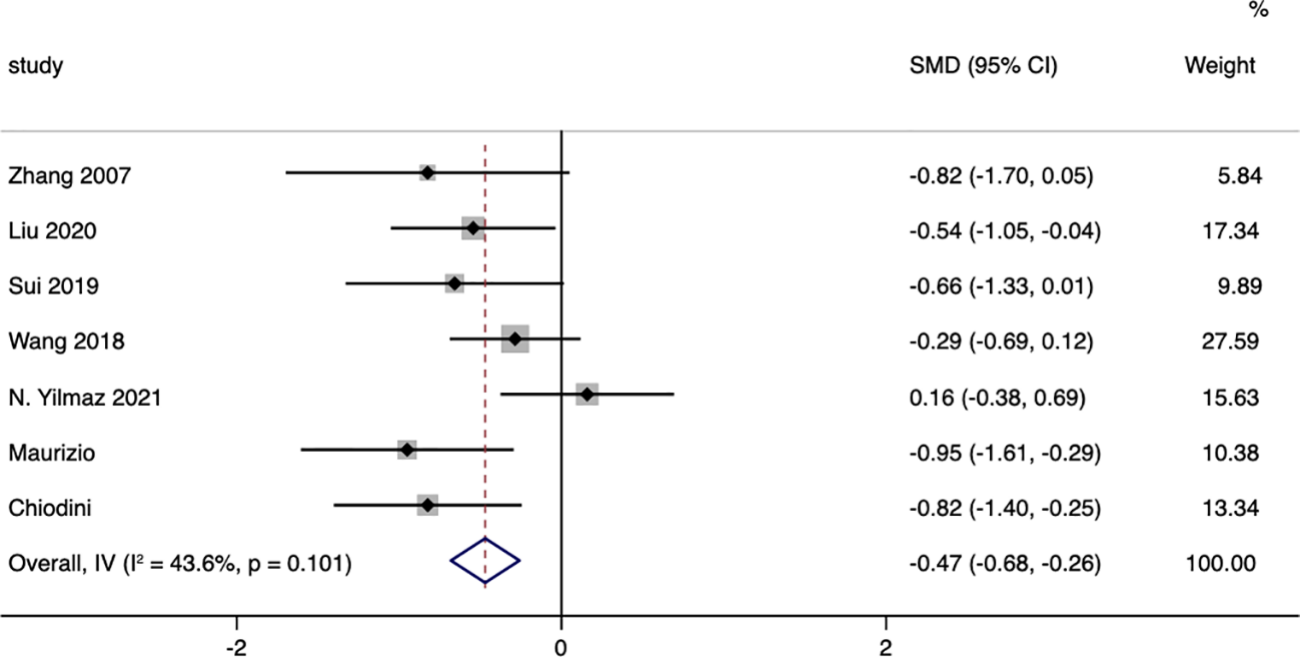
Fasting Blood Glucose-related forest.

Seven studies were included that reported on the changes in fasting blood glucose between surgical patients and those receiving conservative treatment, with I=43.6% and p=0.101, indicating significant heterogeneity among the included studies. Therefore, a random effects model was chosen to pool the effect sizes. The results showed that, compared to conservative treatment, surgery was more effective in reducing patients’ fasting blood glucose (MD= -0.47, 95% CI: -0.68 to -0.26; p < 0.05) (**Figure 4**). The funnel plot (**Supplementary Figure S2**) and Egger’s test (p< 0.05) indicated the presence of publication bias.

**Figure 5:**
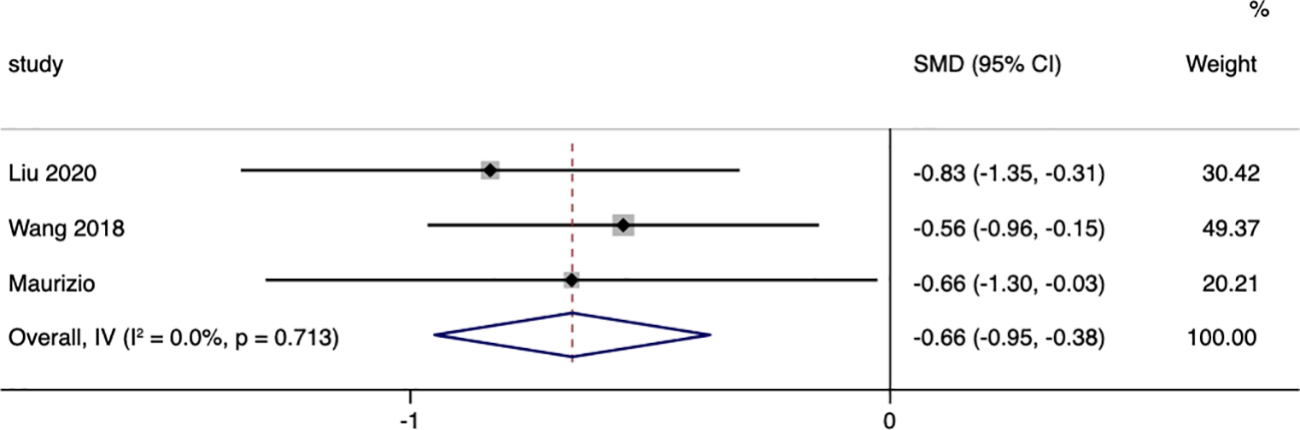
Glycated Hemoglobin (HbA1c)-related forest.

Three studies were included that reported on the changes in glycated hemoglobin (HbA1c) between surgical patients and those receiving conservative treatment, with I=0.0% and p=0.713, indicating very low heterogeneity among the studies. A fixed-effect model was used to pool the effect sizes. The results showed that, compared to conservative treatment, surgery was more effective in reducing patients’ HbA1c (SMD= -0.66, 95% CI: -0.95 to -0.38; p < 0.05) (**Figure 5**).

### Lipid metabolism

**Figure 6:**
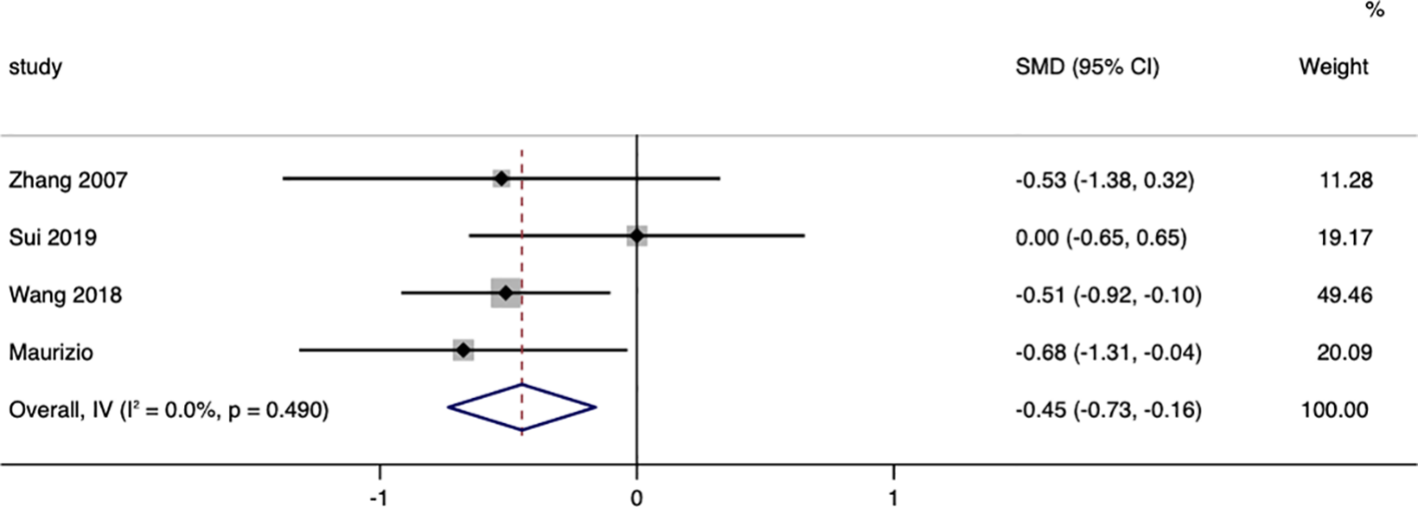
Triglycerides-related forest.

A total of six studies compared the changes in triglycerides between surgical patients and those receiving conservative treatment, with an I=85.0% and p=0.000, indicating a high level of heterogeneity in the literature selection for this study. Upon investigating the sources of heterogeneity and excluding the studies by N. Yilmaz and Liu, a re-evaluation of heterogeneity showed an I=0.0% and p=0.490. Therefore, a random effects model was used to pool the effect sizes. The results demonstrated that surgery could reduce triglycerides in patients, with a standardized mean difference (SMD=-0.45, 95% CI: -0.73 to -0.16; p<0.05) (**Figure 6**). The funnel plot (**Supplementary Figure S3**) and Egger’s test (p=0.260) showed no publication bias.

**Figure 7:**
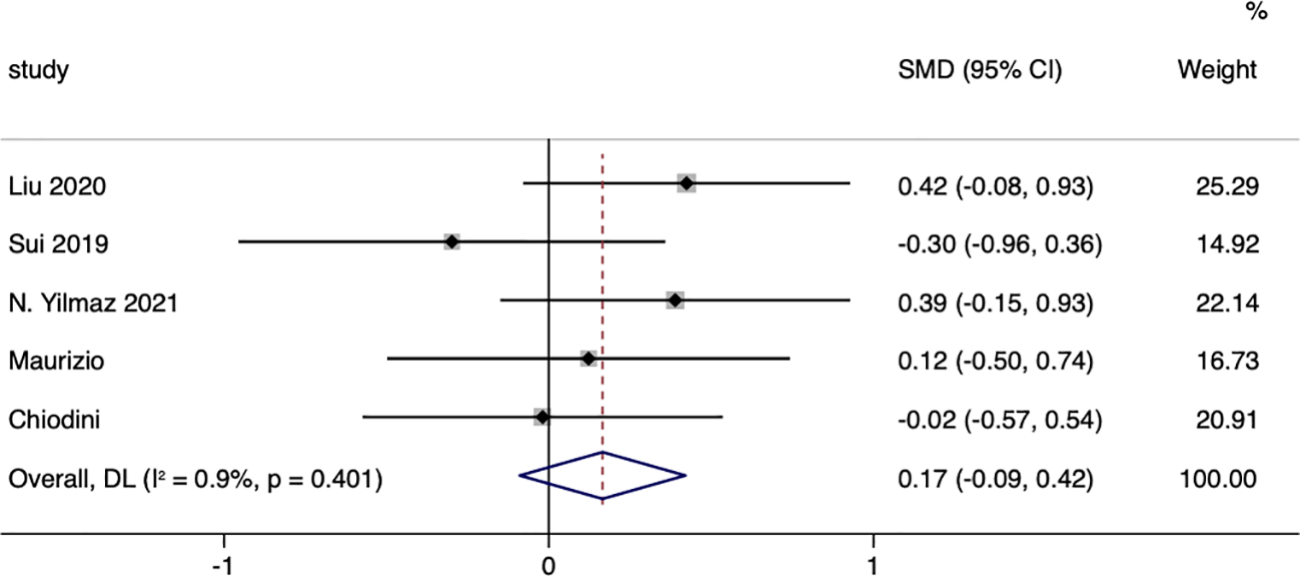
LDL-related fourest.

A total of five studies compared the changes in LDL (low-density lipoprotein) between surgical patients and those receiving conservative treatment, with an I=0.0% and p=0.349, indicating very low heterogeneity in the selection of literature for this study. Therefore, a fixed-effect model was used to pool the effect sizes. The results showed that, compared to the conservative group, surgery did not significantly reduce LDL in patients, with a standardized mean difference (SMD=0.17, 95% CI: -0.09 to 0.42; p=0.203) (**Figure 7**). The funnel plot (**Supplementary Figure S4**) and Egger’s test (p=0.221) showed no publication bias.

### In terms of blood pressure

**Figure 8:**
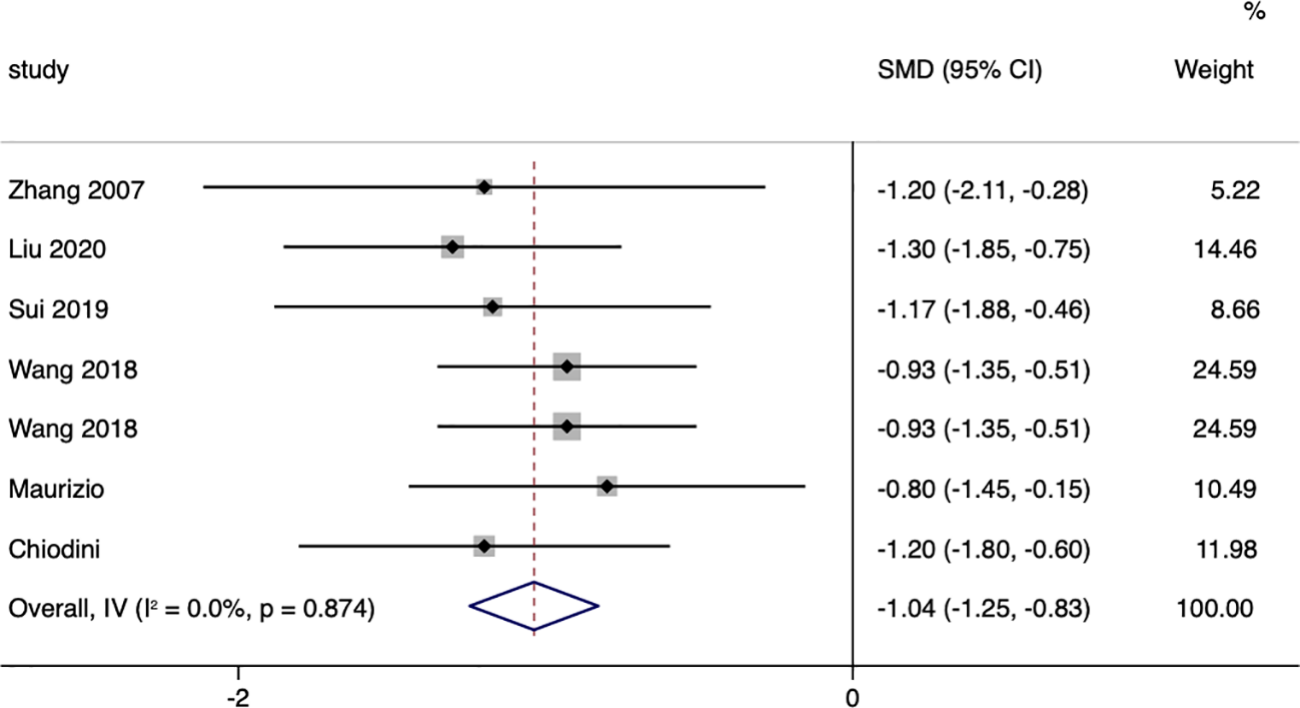
Systolic Blood Pressure-related forest.

In terms of systolic blood pressure, a total of seven studies compared the changes in systolic blood pressure between surgical patients and those receiving conservative treatment, with an I=0.0% and p=0.874, indicating very low heterogeneity in the selection of literature for this study. Therefore, a fixed-effect model was used to pool the effect sizes. The results showed that, compared to the conservative treatment group, surgery was more effective in reducing patients’ systolic blood pressure (SMD= -1.04, 95% CI: -1.25 to -0.83; p<0.05) (**Figure 8**). The funnel plot (**Supplementary Figure S5**) and Egger’s test (p=0.293) showed no publication bias.

**Figure 9:**
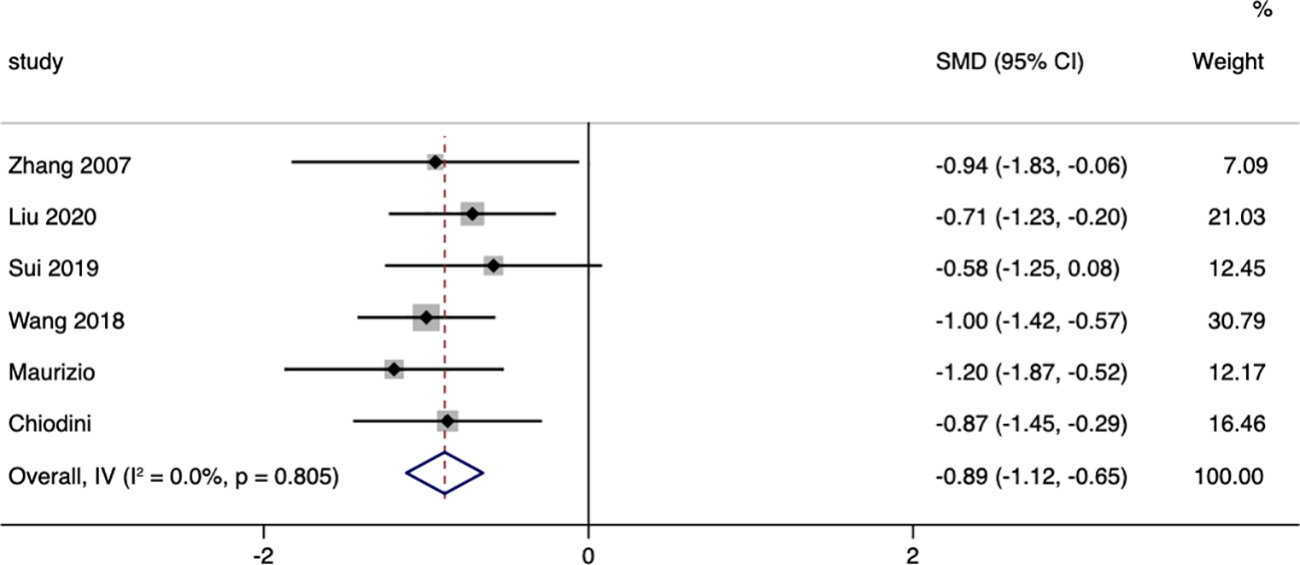
Diastolic Blood Pressure-related forest.

A total of six studies compared the changes in diastolic blood pressure between patients in the surgical group and those receiving conservative treatment, with an I=0.0% and p=0.805, indicating very low heterogeneity in the selection of literature for this study. Therefore, a fixed-effect model was used to pool the effect sizes. The results showed that, compared to the conservative treatment group, surgery was more effective in reducing patients’ diastolic blood pressure, with a standardized mean difference (SMD= -0.89, 95% CI: -1.12 to -0.65; p<0.05) (**Figure 9**). The funnel plot (**Supplementary Figure S6**) and Egger’s test (p< 0.05) indicated the presence of publication bias.

### Hormone levels

**Figure 10:**
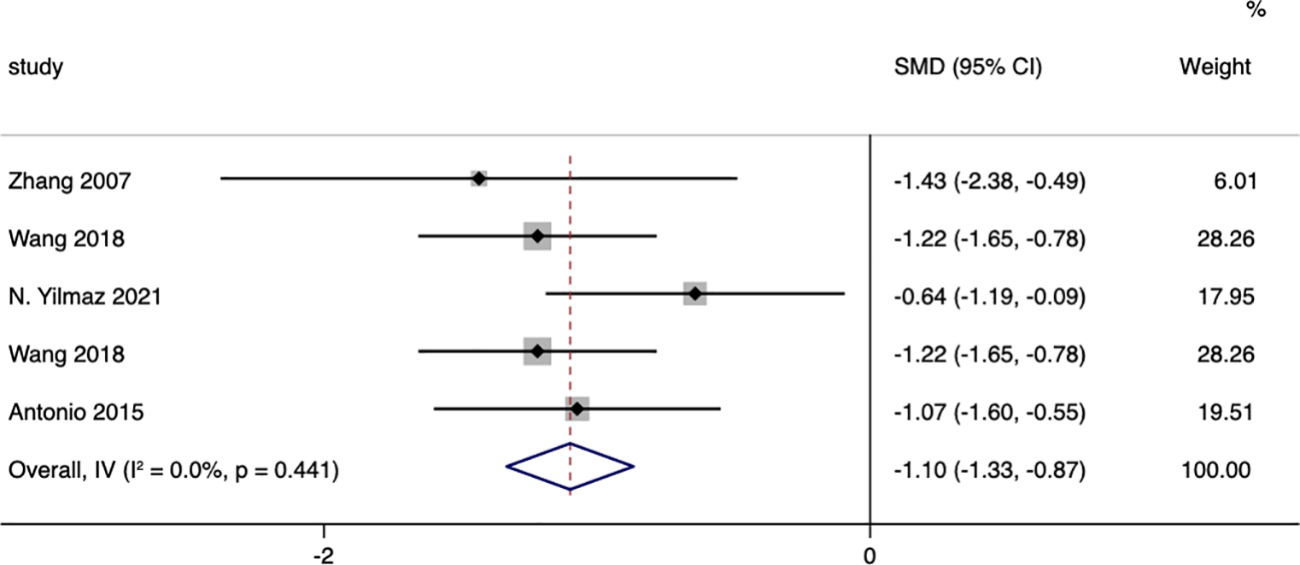
24-hour Urinary Free Cortisol-related forest.

A total of five studies compared the changes in 24-hour urinary free cortisol levels between patients in the surgical group and those receiving conservative treatment, with an I=0.0% and p=0.441, indicating very low heterogeneity in the selection of literature for this study. Therefore, a fixed-effect model was used to pool the effect sizes. The results showed that, compared to the conservative treatment group, surgery resulted in a more significant decrease in patients’ 24-hour urinary free cortisol levels, with a standardized mean difference (SMD= -1.10, 95% CI: -1.33 to -0.87; p<0.05) (**Figure 10**). The funnel plot (**Supplementary Figure S7**) and Egger’s test (p=0.851) showed no publication bias.

**Figure 11:**
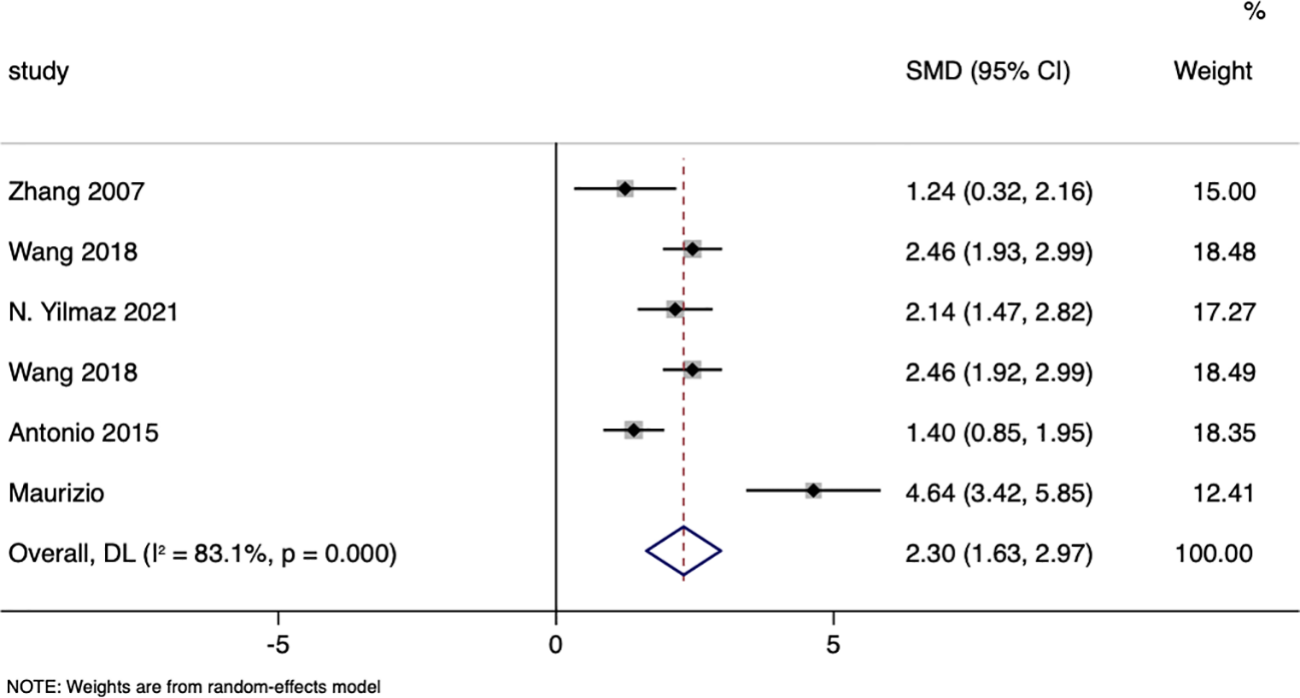
ACTH-related forest.

A total of six studies compared the changes in ACTH (Adrenocorticotropic Hormone) levels between patients in the surgical group and those receiving conservative treatment, with an I=83.1% and p=0.00, indicating a high level of heterogeneity in the selection of literature for this study. Therefore, a random effects model was used to pool the effect sizes. The results showed that, compared to the conservative treatment group, surgery was able to increase patients’ ACTH levels (MD= 2.30,95% CI: 1.63 to 2.97; p<0.05) (**Figure 11**). The funnel plot (**Supplementary Figure S8**) and Egger’s test (p< 0.05) indicated the presence of publication bias.

## Discussion

With the maturation and widespread use of ultrasound and CT imaging technologies, the incidence of adrenal tumors has gradually increased. Among adrenal incidentalomas, approximately 5% to 20% are functional adenomas that produce glucocorticoids (1, 2, 25). Mild Autonomous Cortisol Secretion (MACS) refers to a condition in which the adrenal cortex, often in the context of an adrenal adenoma or other adrenal lesions, autonomously secretes mildly elevated levels of cortisol without stimulation from adrenocorticotropic hormone (ACTH). This condition typically lacks the overt clinical symptoms of Cushing’s syndrome. Studies have shown that patients with MACS are more likely to develop metabolic diseases such as hypertension, type 2 diabetes, obesity, and dyslipidemia compared to patients without cortisol abnormalities. These conditions significantly increase the risk of cardiovascular events and death (11, 26–32).

A 15-year retrospective study analyzing data from 118 patients with adrenal incidentalomas found a significant correlation between MACS and cardiovascular events and mortality. Compared to patients without hormone abnormalities, those with MACS had higher rates of cardiovascular events and mortality (33). The possible pathophysiological mechanisms are: Cortisol increases blood pressure by activating mineralocorticoid receptors, leading to sodium retention and increased blood volume. It also induces insulin resistance, promoting the development of type 2 diabetes, which further elevates cardiovascular risk. Moreover, cortisol dysregulation results in lipid metabolism abnormalities, increasing levels of low-density lipoprotein cholesterol (LDL-C) and triglycerides, which enhance atherosclerotic processes.

Chronic low-grade inflammation is another critical pathophysiological mechanism. Elevated cortisol levels in MACS induce a systemic inflammatory response, marked by increased inflammatory markers such as C-reactive protein and interleukin-6. This inflammation damages endothelial function and promotes arterial stiffness, further exacerbating cardiovascular risk. Additionally, chronic inflammation contributes to endothelial dysfunction, which impairs vascular health and increases the likelihood of cardiovascular events (34, 35).

However, the absence of overt Cushing’s syndrome symptoms makes the condition easy to overlook or misdiagnose, leading to potential under-treatment. Under-treatment can result from the failure to recognize the cardiovascular risks associated with MACS. Without appropriate intervention, these patients remain at elevated risk for adverse cardiovascular outcomes. Our statistical analysis results show that, compared to conservative treatment, surgery can improve glucose and lipid metabolism in patients with MACS, reduce blood pressure and weight, and improve hormone levels, which is similar to the conclusions of some previous studies (29, 36–42). In the management of subclinical Cushing’s syndrome, adrenalectomy as a treatment option has been shown in multiple studies to have significant benefits for patients. Specifically, surgical treatment, compared to conservative management, demonstrates higher efficacy in improving or curing metabolic diseases associated with MACS, such as hypertension, glucose metabolism disorders, and obesity. A systematic review revealed that, in the group of patients who underwent adrenalectomy, 72%, 46%, and 39% of patients achieved cure or significant improvement in blood pressure control, glucose metabolism, and weight management, respectively (43). Studies have suggested that cardiovascular risk factors worsened in patients with MACS in the medically managed group (14, 15, 23, 44). Among the MACS population, patients with osteoporosis have a higher incidence of fractures (45–47), Due to the limitations of the included literature, we were unable to analyze the impact of surgery on osteoporosis in such patients.

Minimally invasive adrenalectomy has been proven to be a safe and effective treatment strategy, demonstrating good clinical outcomes associated with a low risk of complications, even in patients with MACS (48). These research findings highlight the importance and clinical benefits of adopting adrenalectomy for treating MACS under specific circumstances. However, unnecessary adrenalectomy in the treatment of mild autonomous cortisol secretion (MACS) can lead to adverse outcomes due to the ambiguous criteria for surgical intervention. Studies indicate that adverse outcomes post-adrenalectomy include postoperative adrenal insufficiency, persistent hypertension, and the failure to improve certain components of the metabolic syndrome. One study highlighted that a subset of MACS patients undergoing adrenalectomy continued to experience complications such as hypertension and diabetes. Additionally, surgical complications such as bleeding, infection, and postoperative adrenal crisis must be considered. The long-term effects of surgical treatment and lifestyle interventions still require further research and validation (49–51).

Conservative treatment (including pharmacotherapy and lifestyle modifications) offers an alternative treatment approach for patients who are at a higher risk from surgery or who refuse surgical interventions. This method also has the advantages of being less invasive and having a smaller impact on the patient’s daily life, despite its lesser effectiveness in controlling symptoms and reducing complications compared to surgical treatment (52).

Inconsistencies in the diagnostic criteria for patients with MACS lead to heterogeneity in the study populations, which may affect the evaluation of surgical and conservative treatment outcomes. Furthermore, there is still a lack of high-quality, large-scale randomized controlled studies to confirm the best management strategy for patients with MACS. Both surgical and conservative treatments have their advantages and limitations. Choosing the best treatment approach requires a comprehensive consideration of the patient’s specific conditions, preferences, potential risks, and expected treatment outcomes. Future research should focus on comparing the long-term effects of surgical versus conservative treatment and developing personalized treatment strategies to improve the treatment outcomes and quality of life for patients with MACS.

## Data availability statement

The data analyzed in this study is subject to the following licenses/restrictions: The data comes from published studies. Requests to access these datasets should be directed to 709069446@qq.com.

## Author contributions

XR: Writing – original draft. MN: Writing – original draft, Methodology. XZ: Writing – review & editing, Supervision.

## References

[1] Starker LF Kunstman JW Carling T . Subclinical Cushing syndrome: a review. Surg Clin North Am. (2014) 94:657–68. doi: 10.1016/j.suc.2014.02.008 24857582

[2] Chiodini I . Clinical review: Diagnosis and treatment of subclinical hypercortisolism. J Clin Endocrinol Metab. (2011) 96:1223–36. doi: 10.1210/jc.2010-2722 21367932

[3] Czapla-Iskrzycka A Malicka J Zieliński G . Comorbidities in mild autonomous cortisol secretion – A clinical review of literature. Exp Clin Endocrinol Diabetes. (2023) 131:202–10. doi: 10.1055/a-1753-9248 35817047

[4] Ahn SH Song KH Kim J Park S Kim H Cho YY . New diagnostic criteria for subclinical hypercortisolism using postsurgical hypocortisolism: the Co-work of Adrenal Research study. Clin Endocrinol. (2017) 86:10–8. doi: 10.1111/cen.2017.86.issue-1 27341314

[5] Fassnacht M Arlt W Bancos I Dralle H Newell-Price J Sahdev A . Management of adrenal incidentalomas: European Society of Endocrinology Clinical Practice Guideline in collaboration with the European Network for the Study of Adrenal Tumors. Eur J Endocrinol. (2016) 175:G1–G34. doi: 10.1530/EJE-16-0467 27390021

[6] Sherlock M Scarsbrook A Abbas A Fraser S Limumpornpetch P Dineen R . Adrenal incidentaloma. Endocr Rev. (2020) 41(6):775–820. doi: 10.1210/endrev/bnaa008 PMC7431180 32266384

[7] Zografos GN Perysinakis I Vassilatou E . Subclinical Cushing's syndrome: current concepts and trends. Hormones (Athens). (2014) 13:323–37. doi: 10.14310/horm.2002.1506 25079456

[8] Morelli V Ghielmetti A Caldiroli A Grassi S Siri FM Caletti E . Mental health in patients with adrenal incidentalomas: is there a relation with different degrees of cortisol secretion? J Clin Endocrinol Metab. (2021) 106:e130–9. doi: 10.1210/clinem/dgaa695 PMC7765655 33017843

[9] Aresta C Favero V Morelli V Giovanelli L Parazzoli C Falchetti A . Cardiovascular complications of mild autonomous cortisol secretion. Best Pract Res Clin Endocrinol Metab. (2021) 35:101494. doi: 10.1016/j.beem.2021.101494 33814301

[10] Zavatta G Di Dalmazi G . Recent advances on subclinical hypercortisolism. Endocrinol Metab Clin North Am. (2018) 47:375–83. doi: 10.1016/j.ecl.2018.01.003 29754638

[11] Terzolo M Bovio S Reimondo G Pia A Osella G Borretta G . Subclinical Cushing's syndrome in adrenal incidentalomas. Endocrinol Metab Clin North Am. (2005) 34:423–39. doi: 10.1016/j.ecl.2005.01.008 15850851

[12] Akaza I Yoshimoto T Iwashima F Nakayama C Doi M Izumiyama H . Clinical outcome of subclinical Cushing's syndrome after surgical and conservative treatment. Hypertens Res. (2011) 34:1111–5. doi: 10.1038/hr.2011.90 21775997

[13] Toniato A Merante-Boschin I Opocher G Pelizzo MR Schiavi F Ballotta E . Surgical versus conservative management for subclinical Cushing syndrome in adrenal incidentalomas: a prospective randomized study. Ann Surg. (2009) 249:388–91. doi: 10.1097/SLA.0b013e31819a47d2 19247023

[14] Tsuiki M Tanabe A Takagi S Naruse M Takano K . Cardiovascular risks and their long-term clinical outcome in patients with subclinical Cushing's syndrome. Endocr J. (2008) 55:737–45. doi: 10.1507/endocrj.K07E-177 18506093

[15] Chiodini I Morelli V Salcuni AS Eller-Vainicher C Torlontano M Coletti F . Beneficial metabolic effects of prompt surgical treatment in patients with an adrenal incidentaloma causing biochemical hypercortisolism. J Clin Endocrinol Metab. (2010) 95:2736–45. doi: 10.1210/jc.2009-2387 20375210

[16] Kawate H Kohno M Matsuda Y Akehi Y Tanabe M Horiuchi T . Long-term study of subclinical Cushing's syndrome shows high prevalence of extra-adrenal Malignancy in patients with functioning bilateral adrenal tumors. Endocr J. (2014) 61:1205–12. doi: 10.1507/endocrj.EJ14-0155 25223468

[17] Zhang W Tang ZY Wang WG Ning G . Prognosis of subclinical Cushing’s syndrome: comparison of surgical outcomes in patients with or without surgical resection of unexpected tumors. Chin J Endocrinol Metab. (2007) 23:539–40. doi: 10.3760/j.issn:1000-6699.2007.06.023

[18] Liu MS Zhang JW Zhu KY Feng WH Huang H Zhu DL . The clinical characteristics and comparison of prognosis between surgical and conservative treatment in subclinical Cushing's syndrome. Chin Med J. (2020) 100:2834–40. doi: 10.3760/cma.j.cn112137-20200213-00274 32988143

[19] Sui H Chen HL Zhao JL Zhou YH Geng XQ . Changes of bone mineral density and biochemical indexes after surgery in subclinical hypercortisolism patients with adrenal incidentaloma. J Community Med. (2019) 17:459–62.

[20] Wang D Zhang YS Chen Li HZ . Surgical treatment of subclincal Cushing syndrome. Chin J Urol. (2017) 41(6):e130–9.

[21] Yilmaz N Tazegul G Sari R Avsar E Altunbas H Balci MK . Effectiveness of unilateral adrenalectomy in bilateral adrenal incidentaloma patients with subclinical hypercortisolemia. Acta Endocrinol (Buchar). (2021) 17:479–85. doi: 10.4183/aeb.2021.479 PMC9206144 35747873

[22] Wang D Ji ZG Li HZ Zhang YS . Adrenalectomy was recommended for patients with subclinical Cushing's syndrome due to adrenal incidentaloma. Cancer biomark. (2018) 21:367–72. doi: 10.3233/CBM-170531 PMC13078276 29125476

[23] Iacobone M Citton M Viel G Boetto R Bonadio I Mondi I . Adrenalectomy may improve cardiovascular and metabolic impairment and ameliorate quality of life in patients with adrenal incidentalomas and subclinical Cushing's syndrome. Surgery. (2012) 152:991–7. doi: 10.1016/j.surg.2012.08.054 23158173

[24] Salcuni AS Morelli V Eller Vainicher C Palmieri S Cairoli E Spada A . Adrenalectomy reduces the risk of vertebral fractures in patients with monolateral adrenal incidentalomas and subclinical hypercortisolism. Eur J Endocrinol. (2016) 174:261–9. doi: 10.1530/EJE-15-0977 26630908

[25] Reincke M . Subclinical cushing's syndrome. Endocrinol Metab Clin North Am. (2000) 29:43–56. doi: 10.1016/S0889-8529(05)70115-8 10732263

[26] Giordano R Marinazzo E Berardelli R Picu A Maccario M Ghigo E . Long-term morphological, hormonal, and clinical follow-up in a single unit on 118 patients with adrenal incidentalomas. Eur J Endocrinol. (2010) 162:779–85. doi: 10.1530/EJE-09-0957 20103607

[27] Terzolo M Bovio S Pia A Conton PA Reimondo G Dall'Asta C . Midnight serum cortisol as a marker of increased cardiovascular risk in patients with a clinically inapparent adrenal adenoma. Eur J Endocrinol. (2005) 153:307–15. doi: 10.1530/eje.1.01959 16061838

[28] Tauchmanovà L Rossi R Biondi B Pulcrano M Nuzzo V Palmieri EA . Patients with subclinical Cushing's syndrome due to adrenal adenoma have increased cardiovascular risk. J Clin Endocrinol Metab. (2002) 87:4872–8. doi: 10.1210/jc.2001-011766 12414841

[29] Emral R Uysal AR Asik M Gullu S Corapcioglu D Tonyukuk V . Prevalence of subclinical Cushing's syndrome in 70 patients with adrenal incidentaloma: clinical, biochemical and surgical outcomes. Endocr J. (2003) 50:399–408. doi: 10.1507/endocrj.50.399 14599113

[30] Chiodini I Albani A Ambrogio AG Campo M De Martino MC Marcelli G . Six controversial issues on subclinical Cushing's syndrome. Endocrine. (2017) 56:262–6. doi: 10.1007/s12020-016-1017-3 27406391

[31] Di Dalmazi G Vicennati V Rinaldi E Morselli-Labate AM Giampalma E Mosconi C . Progressively increased patterns of subclinical cortisol hypersecretion in adrenal incidentalomas differently predict major metabolic and cardiovascular outcomes: a large cross-sectional study. Eur J Endocrinol. (2012) 166:669–77. doi: 10.1530/EJE-11-1039 22267278

[32] Pelsma IC Fassnacht M Tsagarakis S Terzolo M Tabarin A Sahdev A . Comorbidities in mild autonomous cortisol secretion and the effect of treatment: systematic review and meta-analysis. Eur J Endocrinol. (2023) 189:S88–101. doi: 10.1093/ejendo/lvad134 37801655

[33] Di Dalmazi G Vicennati V Garelli S Casadio E Rinaldi E Giampalma E . Cardiovascular events and mortality in patients with adrenal incidentalomas that are either non-secreting or associated with intermediate phenotype or subclinical Cushing's syndrome: a 15-year retrospective study. Lancet Diabetes Endocrinol. (2014) 2:396–405. doi: 10.1016/S2213-8587(13)70211-0 24795253

[34] Ueland G Methlie P Heie A Meling Stokland AE Dahle AL Sævik ÅB . Substantial changes in inflammatory and cardiovascular biomarkers in patients with autonomous cortisol secretion. Eur J Endocrinol. (2023) 189:78–86. doi: 10.1093/ejendo/lvad076 37421314

[35] Dimitriadis GK Kaur J Mytilinaiou M Davasgaium A Sambrook D Hewins C . Nuclear factor-kappa beta activation and monocyte-endothelial adhesion lead to chemerin induced endothelial cell inflammation. Endocrine Abstracts. (2017) 50. doi: 10.1530/endoabs.50.P211

[36] Debono M Bradburn M Bull M Harrison B Ross RJ Newell-Price J . Cortisol as a marker for increased mortality in patients with incidental adrenocortical adenomas. J Clin Endocrinol Metab. (2014) 99:4462–70. doi: 10.1210/jc.2014-3007 PMC4255126 25238207

[37] Bae JC . Subclinical cushing's syndrome and metabolic disorder. Endocrinol Metab (Seoul). (2014) 29:441–2. doi: 10.3803/EnM.2014.29.4.441 PMC4285047 25559573

[38] Fernández-Real JM Engel WR Simó R Salinas I Webb SM . Study of glucose tolerance in consecutive patients harbouring incidental adrenal tumours. Study Group of Incidental Adrenal Adenoma. Clin Endocrinol (Oxf). (1998) 49:53–61. doi: 10.1046/j.1365-2265.1998.00437 9797847

[39] Saadulla A Abbas A Sagar R . Evidence of mild autonomous cortisol secretion in patients with adrenal incidentaloma is associated with increased cardiometabolic morbidity and relative risk of cardiovascular disease, compared to those with non-functional adrenal incidentalomas. Endocrine Abstracts. (2023) 94. doi: 10.1530/endoabs.94.P9

[40] Saadulla A Sagar R Wadsworth C Cooke H Abbas A . Initial impact of a virtual pathway to evaluate patients with evidence of mild autonomous cortisol secretion. Endocrine Abstracts. (2023) 94. doi: 10.1530/endoabs.94.P6

[41] Reincke M Lamas C . Epidemiology and management of hypertension and diabetes mellitus in patients with mild autonomous cortisol secretion: a review. Biomedicines. (2023) 11:3115. doi: 10.3390/biomedicines11123115 38137336 PMC10740610

[42] Bancos I Alahdab F Crowley RK Chortis V Delivanis DA Erickson D . Improvement of cardiovascular risk factors after adrenalectomy in patients with adrenal tumors and subclinical Cushing's syndrome: a systematic review and meta-analysis. Eur J Endocrinol. (2016) 175:R283–95. doi: 10.1530/EJE-16-0465 27450696

[43] Iacobone M Citton M Scarpa M Viel G Boscaro M Nitti D . Systematic review of surgical treatment of subclinical Cushing's syndrome. Br J Surg. (2015) 102:318–30. doi: 10.1002/bjs.9742 25640696

[44] Petramala L Cavallaro G Galassi M Marinelli C Tonnarini G Concistrè A . Clinical benefits of unilateral adrenalectomy in patients with subclinical hypercortisolism due to adrenal incidentaloma: results from a single center. High Blood Press Cardiovasc Prev. (2017) 24:69–75. doi: 10.1007/s40292-017-0182-7 28138953

[45] Di Dalmazi G Pasquali R Beuschlein F Reincke M . Subclinical hypercortisolism: a state, a syndrome, or a disease? Eur J Endocrinol. (2015) 173(4):61–71. doi: 10.1530/EJE-15-0272 26282599

[46] Chiodini I Torlontano M Carnevale V Guglielmi G Cammisa M Trischitta V . Bone loss rate in adrenal incidentalomas: a longitudinal study. J Clin Endocrinol Metab. (2001) 86:5337–41. doi: 10.1210/jcem.86.11.8022 11701701

[47] Morelli V Eller-Vainicher C Salcuni AS Coletti F Iorio L Muscogiuri G . Risk of new vertebral fractures in patients with adrenal incidentaloma with and without subclinical hypercortisolism: a multicenter longitudinal study. J Bone Miner Res. (2011) 26:1816–21. doi: 10.1002/jbmr.398 21472775

[48] Hsieh LB Mackinney E Wang TS . When to intervene for subclinical cushing's syndrome. Surg Clin North Am. (2019) 99:747–58. doi: 10.1016/j.suc.2019.04.011 31255204

[49] Corbetta S Dall’Asta C Mantovani G Ferrero S Castellano M Patrone C . Comorbidities in mild autonomous cortisol secretion and the effect of adrenalectomy: a randomized controlled trial. Eur J Endocrinol. (2023) 189:G1–G20. doi: 10.1530/EJE-23-0290

[50] Pivonello R De Martino MC Negri M Simeoli C De Leo M Colao A . Adrenalectomy improves blood pressure and metabolic control in patients with possible autonomous cortisol secretion: Results of a randomized controlled trial. Front Endocrinol (Lausanne). (2023) 14:728610. doi: 10.3389/fendo.2023.728610

[51] Ermetici F Malavazos AE Monti V Masi S Mancia G . Assessment of mild autonomous cortisol secretion among incidentally discovered adrenal masses: The impact on hypertension and metabolic outcomes. J Clin Endocrinol Metab. (2023) 108:132–42. doi: 10.1210/clinem/dgab723

[52] Li X Zhan X Yu Y Xi H . For small (1-3cm) nonfunctional adrenal incidentaloma (NFAI), which option is more appropriate for conservative treatment or surgery? Front Endocrinol (Lausanne). (2023) 14:728610. doi: 10.3389/fendo.2023.728610 PMC9929361 36817594

