# Supplementary material for: Evaluating the efficacy of surgical and conservative approaches in mild autonomous cortisol secretion: a meta-analysis
Source: Front Endocrinol (Lausanne). 2024 Jul 17;15:1399311. doi: 10.3389/fendo.2024.1399311 (PMC11288901; doi:10.3389/fendo.2024.1399311)
Supplement: Supplementary file 2 [file DataSheet_2.doc]

1.heterogeneity analysis,fixed effects meta-analysis

metan expn expmean expsd cotrn cotrmean cotrsd, fixed label(namevar= study )

2.random effects meta-analysis

metaan _ES _seES,label( study ) dl forest

3.random effects significance tes

metareg _ES,wsse( _seES ) eform

4.ensitivity analysis"

metainf _ES _seES,id( study )
