# Supplementary material for: Evaluating the efficacy of surgical and conservative approaches in mild autonomous cortisol secretion: a meta-analysis
Source: Front Endocrinol (Lausanne). 2024 Jul 17;15:1399311. doi: 10.3389/fendo.2024.1399311 (PMC11288901; doi:10.3389/fendo.2024.1399311)

Supplementary Figure 1.BMI-related funnel plot.


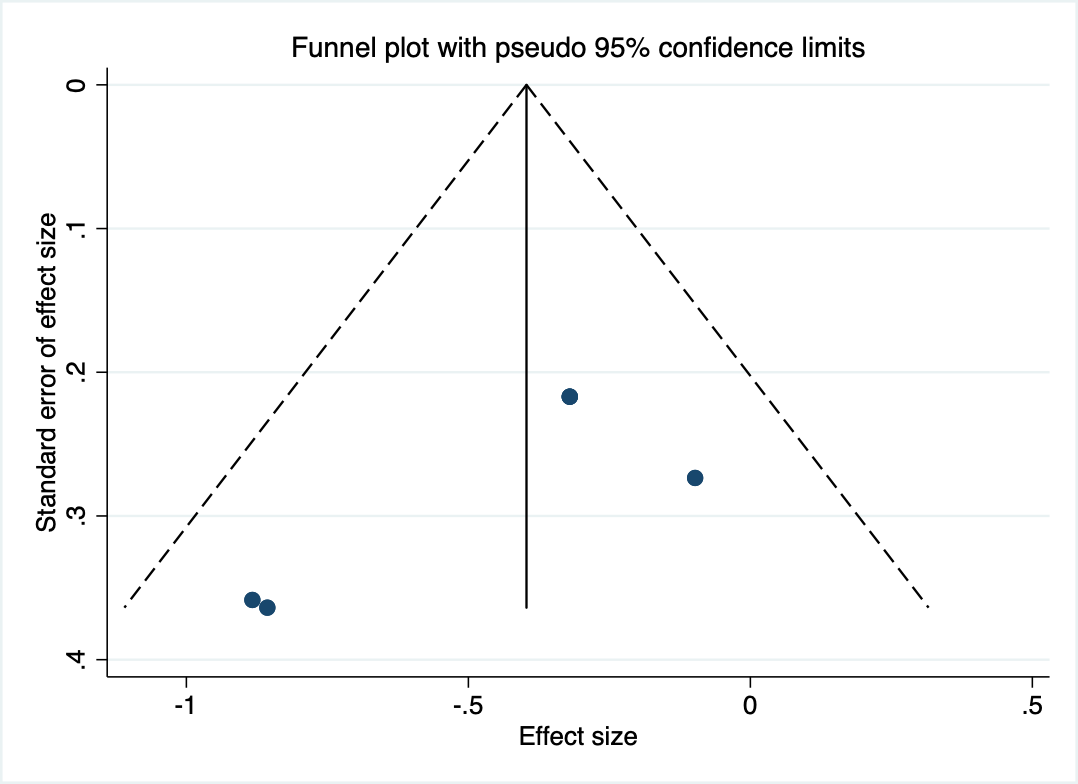


Supplementary Figure 2.FBG-related funnel plot.


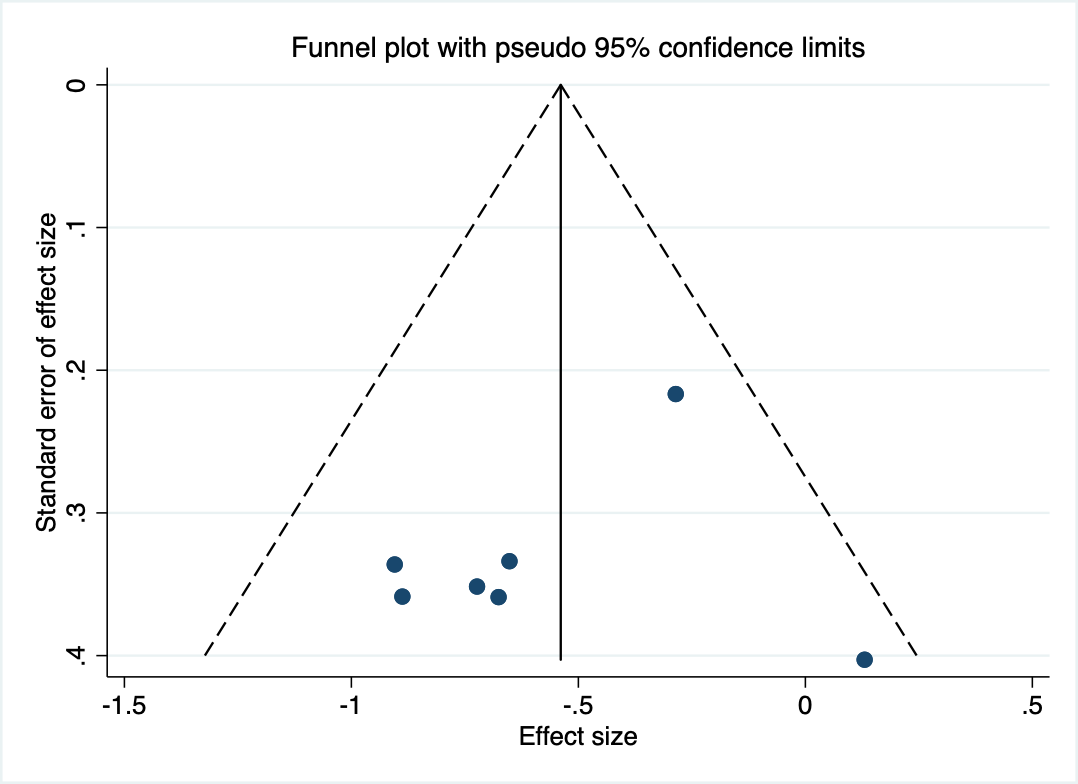


Supplementary Figure 3.triglycerides-related funnel plot.


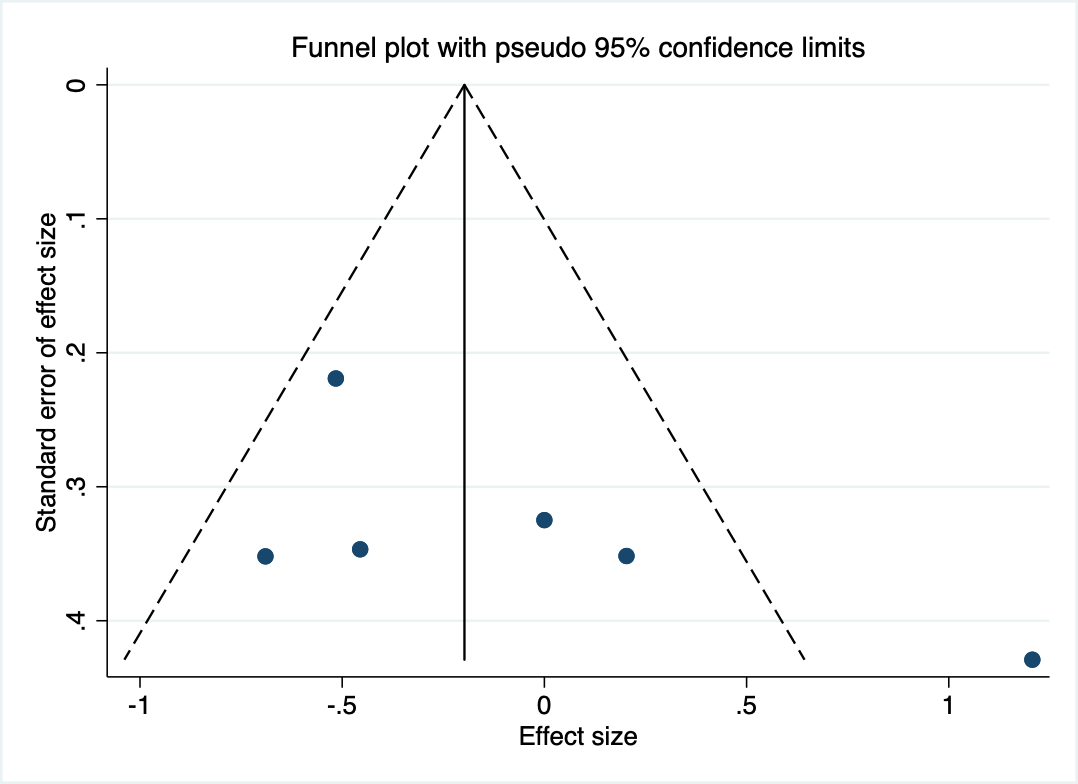


Supplementary Figure 4.LDL-related funnel plot.


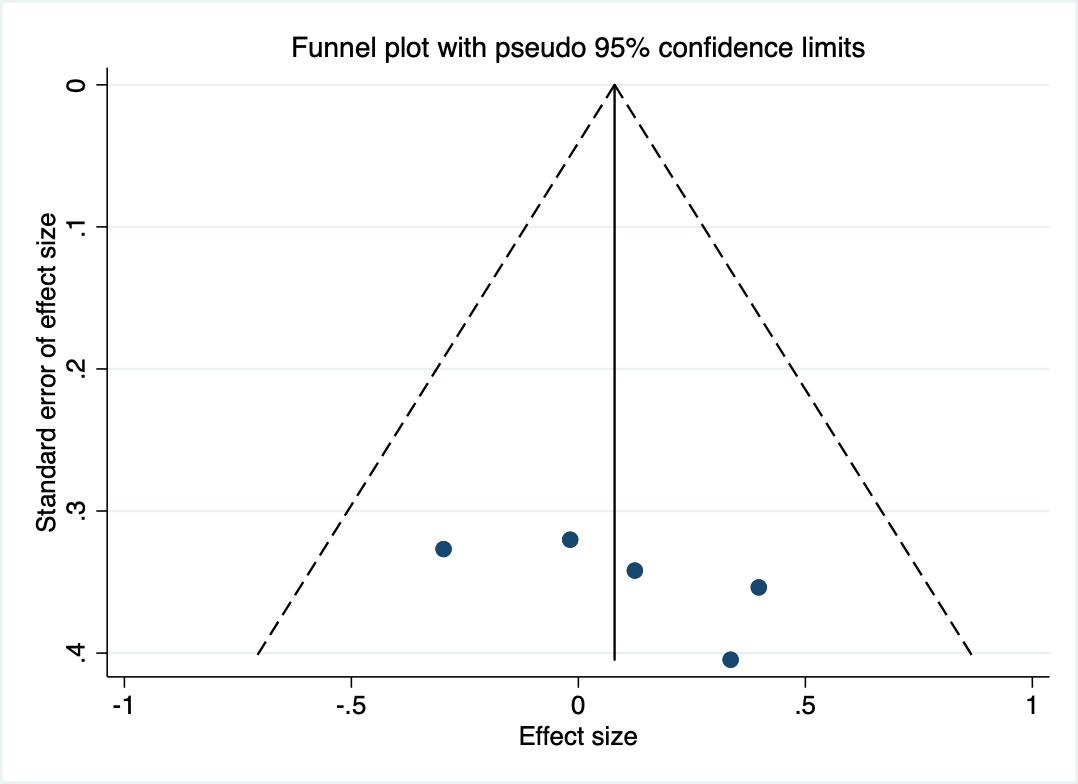


Supplementary Figure 5.SBP-related funnel plot.


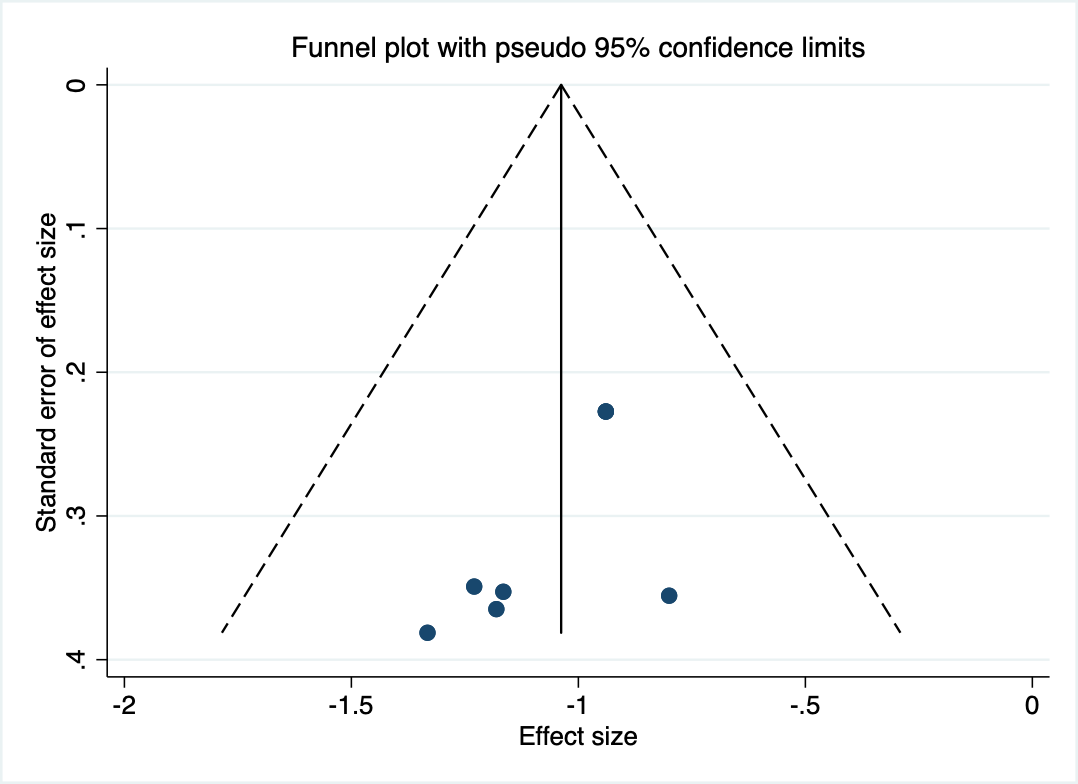


Supplementary Figure 6.DBP-related funnel plot.


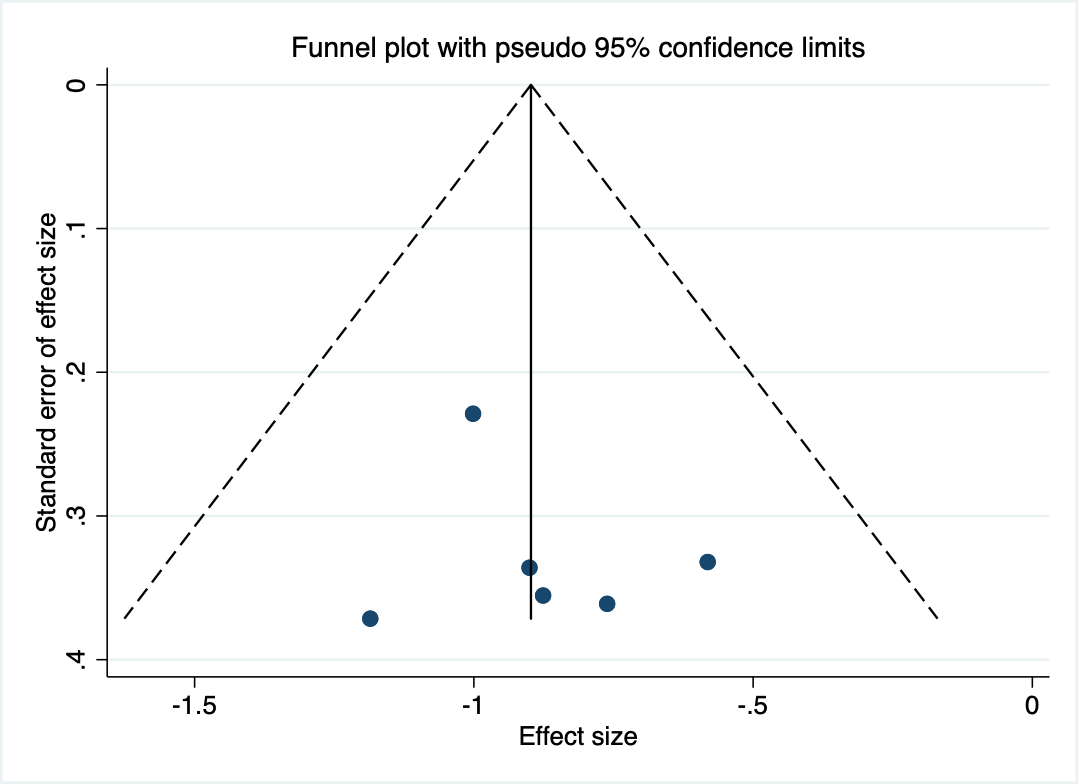


Supplementary Figure 7.24-hour urinary free cortisol levels-related funnel plot.


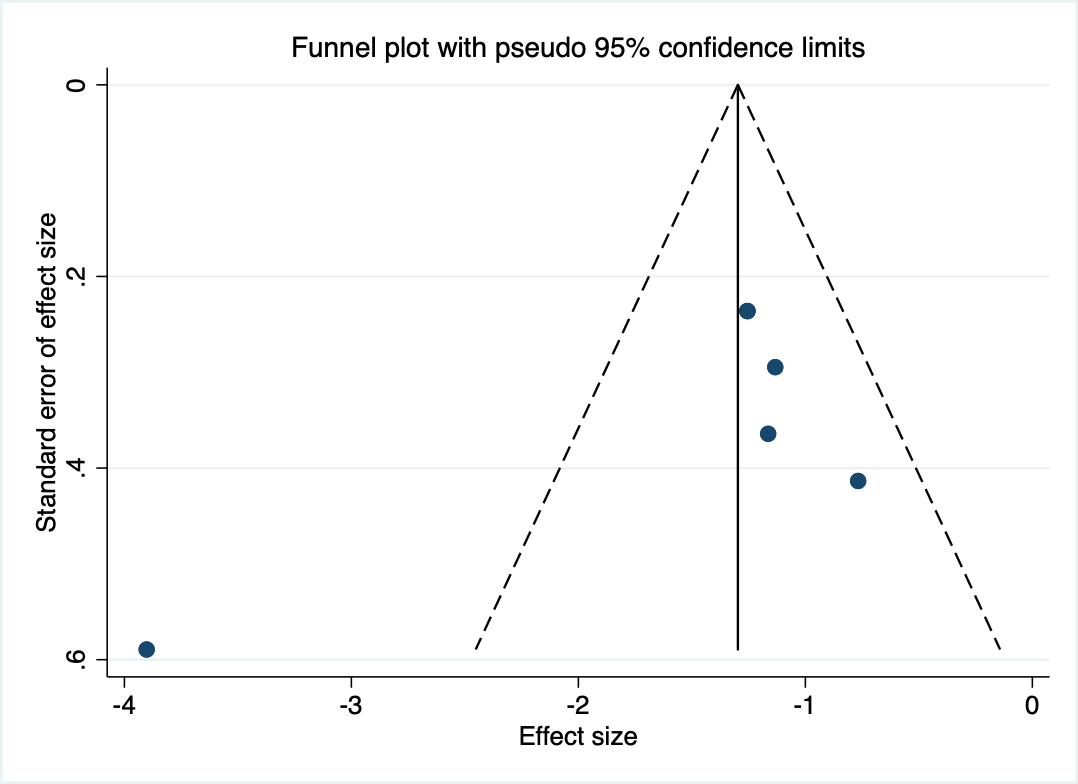


Supplementary Figure 8.ACTH-related funnel plot.


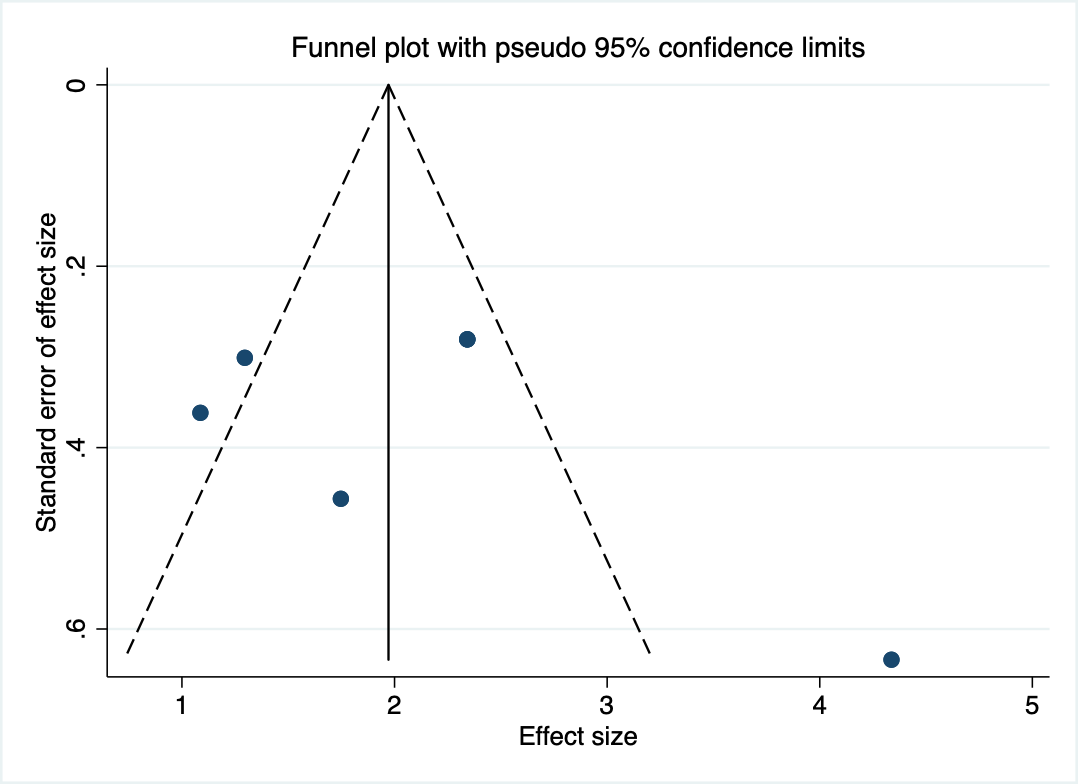

Supplement: Supplementary file 3 [file DataSheet_3.docx]
